# SARS-CoV-2: Emergence of New Variants and Effectiveness of Vaccines

**DOI:** 10.3389/fcimb.2021.777212

**Published:** 2021-12-14

**Authors:** Desh Deepak Singh, Amna Parveen, Dharmendra Kumar Yadav

**Affiliations:** ^1^ Amity Institute of Biotechnology, Amity University Rajasthan, Jaipur, India; ^2^ Gachon Institute of Pharmaceutical Science and Department of Pharmacy, College of Pharmacy, Gachon University, Incheon, South Korea

**Keywords:** SARS-CoV-2, variant, vaccine, neutralization, infectivity

## Abstract

The emergence of SARS-CoV-2 variants may cause resistance at the immunity level against current vaccines. Some emergent new variants have increased transmissibility, infectivity, hospitalization, and mortality. Since the administration of the first SARS-CoV-2 vaccine to a human in March 2020, there is an ongoing global race against SARS-CoV-2 to control the current pandemic situation. Spike (S) glycoprotein of SARS-CoV-2 is the main target for current vaccine development, which can neutralize the infection. Companies and academic institutions have developed vaccines based on the S glycoprotein, as well as its antigenic domains and epitopes, which have been proven effective in generating neutralizing antibodies. The effectiveness of SARS-CoV-2 vaccines and other therapeutics developments are limited by the new emergent variants at the global level. We have discussed the emergent variants of SARS-CoV-2 on the efficacy of developed vaccines. Presently, most of the vaccines have been tremendously effective in severe diseases. However, there are still noteworthy challenges in certifying impartial vaccines; the stories of re-infections are generating more stressful conditions, and this needs further clinical evaluation.

## 1 Introduction

The emergence globally of multiple variants of concern (VOCs) may cause greater severity of infection and transmissibility (Abouelela et al., 2021). Neutralization effects reduced by antibodies are attained *via* naturally occurring infection or vaccination and decrease the effectiveness of vaccines or therapeutics options. Classification systems for genetic variants have been established by the CDC (Center for Disease Control and Prevention) and the WHO (World Health Organization) independently for distinguishing the emerging VOCs and variants of interest (VOIs). The VOCs are classified by the WHO as Alpha (B.1.1.7), Beta (B.1.351), Gamma (P.1), and Delta (B.1.617.2) (Wang et al., 2021). All These strains have shown genetic modification in the S gene in comparison with the native Wuhan strain. The maximum number of mutations in S protein change the infection rate, severity, affinity with host receptor ACE2, and also the possibility to alter the effectiveness of neutralizing antibodies and vaccine efficacy. B.1.351 variants of SARS-CoV-2 have been identified in more than 40 countries at the global level, P.1 variants have been identified in 20 countries (Weisblum et al., 2020). Most of the vaccines are produced for the early strain circulating at the global level. Therefore, some vaccines had reduced efficacy against the newly emerged SARS-CoV-2 variants. Nevertheless, The US FDA (Food and Drug Administration) stated that all FDA-approved vaccinations are still effective against circulating SARS-CoV-2 strains as of February 2021. Here, we will discuss SARS-CoV-2 New Variants and characteristic features and impact on the efficacy of different vaccines to understand their role in the transmissible and vaccine effectiveness.

## 2 Phylogenetic Analysis of SARS-CoV-2 Genome

**Figure 1:**
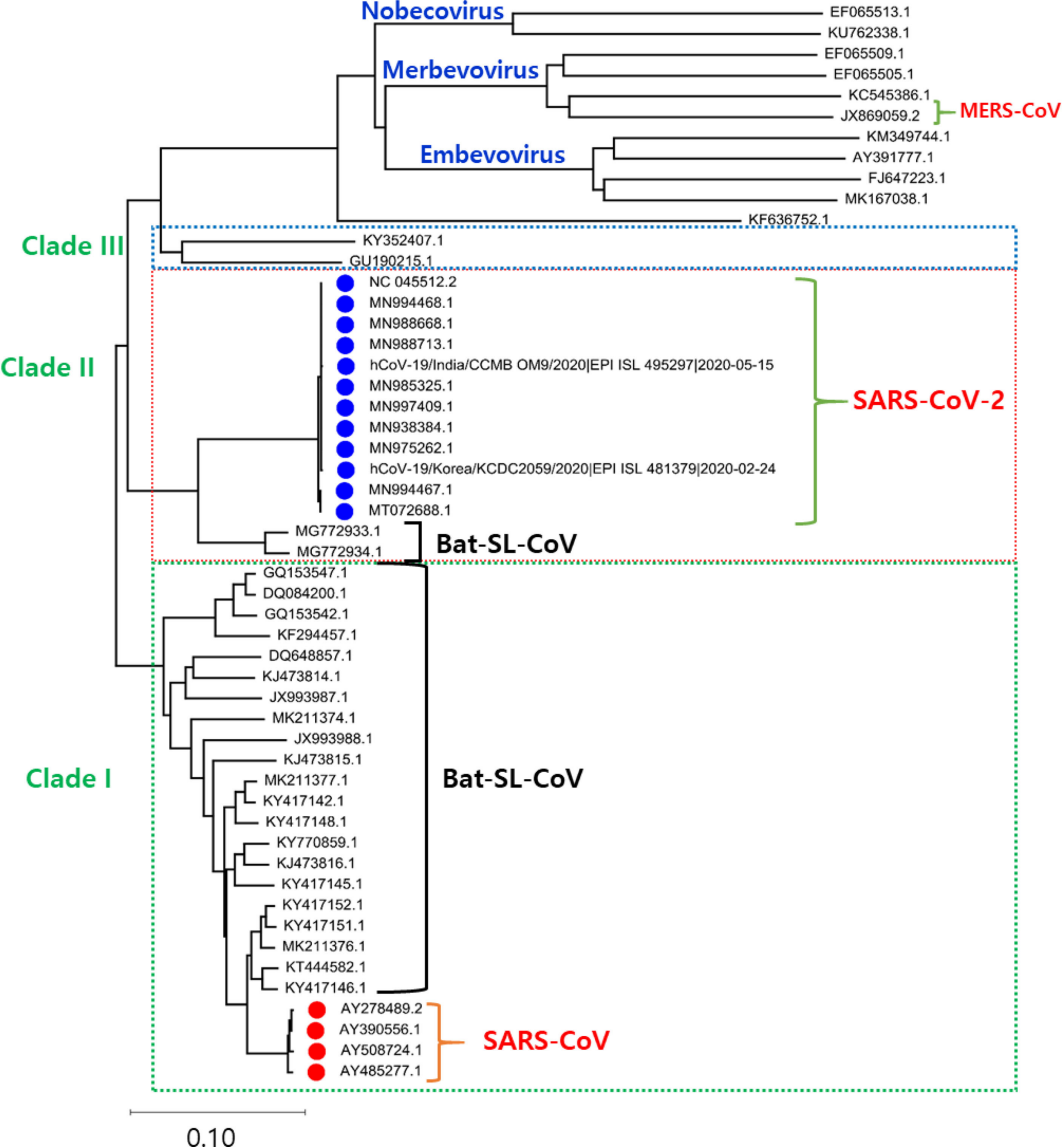
The phylogenetic tree is generated using the latest complete genome sequences of different neighbours, MERS-CoV, Bat-SL-CoV, and SARS-CoV. The tree is divided into three clades according to the grouping of clusters. In Clade I: Bat-SL-CoV-2 and SARS-CoV were showing a close evolutionary relationship with each other. Clade II: A mixture of human and bat coronavirus including the MERS-CoV. Clade III: This clade represents all the SARS-CoV2 genomes isolated from humans, interestingly it is also observed that these genomes are showing a close evolutionary relationship with Bat-SL-CoV-2.

The Phylogenetic analysis of the SARS-CoV-2 genome with the related family members of SARS-CoV-2 from various organisms disclose that the genome of SARS-CoV-2 shows a high evolutionary association with Bat-SL-CoV. The phylogenetic tree is classified into three clades. Clade I consist of Bat-SL-CoV genomes and SARS-CoV with shared sequence identity ranging from more than 80% to 98%. Clade III consists of 11 complete genomes of a mixed form of coronavirus and MERS-CoV-2 genomes and shares the sequence identity from more than 75% to 85%. Clade II consists of 12 SARS-CoV-2 from India (CCMBOM9/2020/EPI ISL 495297), Korea (KCDC2059/2020/EPI ISL 481379), and two Bat-SL-CoV complete genomes and share sequence identity range from 85% to 99%, mainly the SARS-CoV-2 genomes isolated from human samples show a sequence identity range from 98% to 100%. In the analysis of the genome, there is no major divergence was observed in different countries as shown in **Figure 1**.

## 3.SARS-CoV-2 Vaccine and Variants

### 3.1 SARS-CoV-2 Vaccine

**Figure 2:**
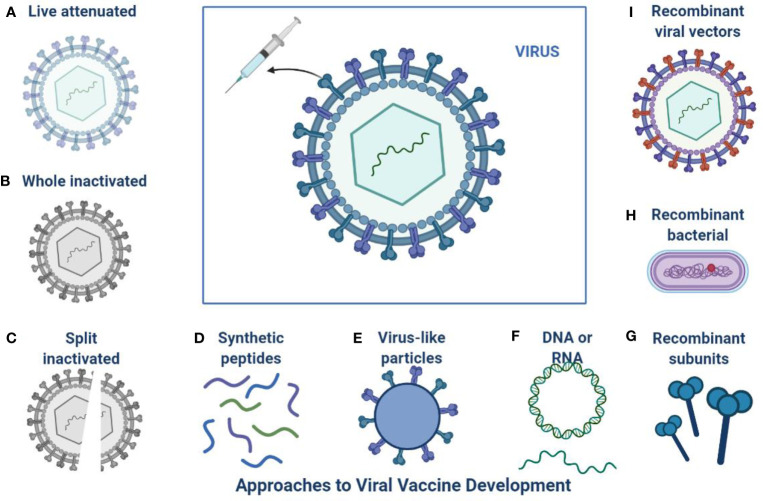
Approaches to SARS-CoV-2 Vaccine development: This is an overview of different platforms for viral vaccine development including **(A)** Live attenuated **(B)** Whole inactivated **(C)** Split inactivated **(D)** Use of synthetic peptides **(E)** Virus like particles **(F)** DNA or RNA **(I)** Recombinant viral vectors **(H)** Recombinant bacterial **(G)** Recombinant subunits.

The current pandemic situation is ongoing and a continuous threat to public health, and still, no anti- SARS-CoV-2 drugs or vaccine options have shown absolute health benefits (Singh et al., 2020a; Singh et al., 2020b). SARS-CoV-2 is very challenging due to age factors, gender differences, ecological factors, and its quick evolution (de Oliveira et al., 2021). The investigations from various fields to come up with effective treatment options and vaccine developments are shown in **Figure 2** (Marian et al., 2021). The challenge of finding a final targeted drug is still difficult and ongoing, 405 therapeutic drugs are under investigation in various clinical stages and 242 vaccines are under clinical 139 research (Park et al., 2021).

Effective vaccines are required against the infection of SARS-CoV-2 for lifelong immunity, and various types of vaccines are under clinical investigation (**Table 1**) such as nucleic acids, lipid-coated, mRNA, peptides, live or attenuated vaccines, and adenovirus-based anti-SARS-CoV-2 (Kyriakidis et al., 2021; Lazarus et al., 2021). The efficacy of SARS-CoV-2 vaccine clinical trials is shown in **Table 1**. Approximately 17 vaccines are in Phase I and 23 vaccines are in Phase-I-II, while 20 have reached stage III for clinical evaluation, and 10 various types of vaccines are approved by different regulatory agencies for community use. CanSino Biologics has developed a Vector-based S protein vaccine and efficacy was evaluated in 603 volunteers and observed effective humoral immune response (Hou et al., 2021). A high level of antibodies production was analyzed after the booster dose. Inactivated and whole vaccines are under clinical examination and 320 Individuals have established effective neutralizing antibodies (Khoury et al., 2021).

**Table 1:** Efficacy of SARS-CoV-2 vaccine on Clinical trials.

SARS- CoV-2 variant	Key mutations	First detected	Transmissibility	Vaccine-mediated protection						
				Corona Vac (Sinovac)	NVX- CoV2373 (Novavax)	mRnA-1273 (moderna)	Ad26.CoV2-S (Johnson & Johnson)	AZD1222 (AstraZeneca– university of oxford)	BnT162b2 (Pfizer– BionTech)	References
Wuhan strain	Reference strain	China, December 2019	Original strain	50–90%	89%	94.1%	66%	55–81%	95%	Rahimi et al., 2021
B.1.617.2 (Delta)	L452R	India, December 2020	97% increase	Not known	Not known	Neutralization titer 6.8-l	Reports of 60% effectiveness	92% effective against hospitalization	one dose of vaccine is 88% effective	Tregoning et al., 2021
	T478K									
	D614G									
	P681R									
Alpha, B.1.1.7	H69/V70 deleti	UK, September 2020	~50% increase	Unknown	86%	2.3–6.4 in titres of neutralizing antibodies	70%	75%	90%	Wang et al., 2021; Abu-Raddad et al., 2021
Beta, B.1.351	D614G	South	25% increase	Unknown	60%	Reduced levels of neutralizing antibodies	72% efficacy in the USA, and 57% in South Africa	10%	75%	Hoffmann et al., 2021
	K417N	Africa,								
	E484K	September 2020								
	N501Y									
Gamma, P.1 (B.1.1.28.1)	E484K	Japan/	1.4–2.2 times more transmissible	51%	Unknown	Reduced levels of neutralizing antibodies	68%	Unknown	No evidence of reduced protection	Aleem et al., 2021
	K417N/T	Brazil,								
	N501Y	December 2020								
	D614G									

### 3.2 SARS-CoV-2 Variants

**Figure 3:**
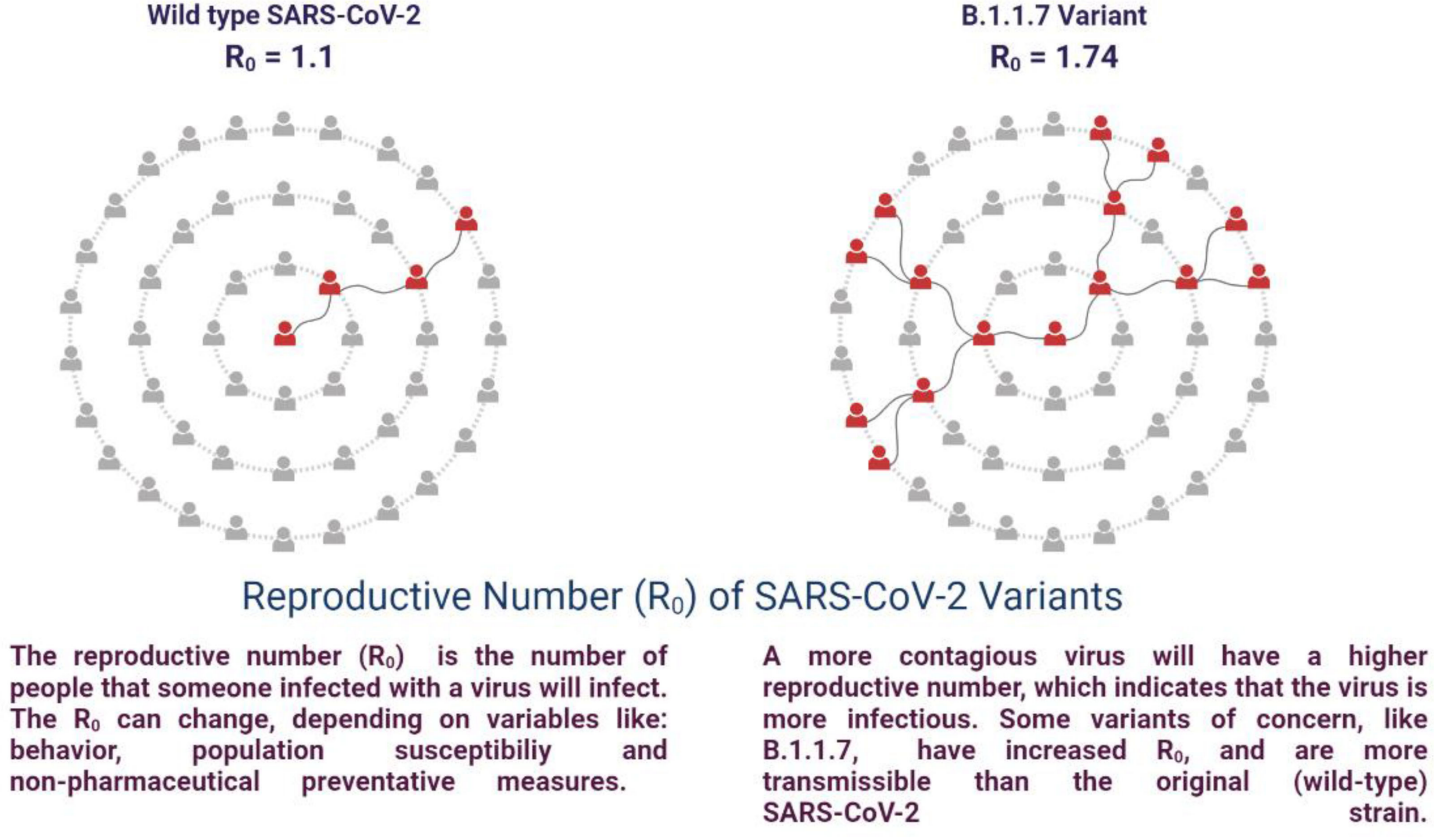
Potential transmission of the mechanism of SARS-CoV-2 variants of Concern.

**Figure 4:**
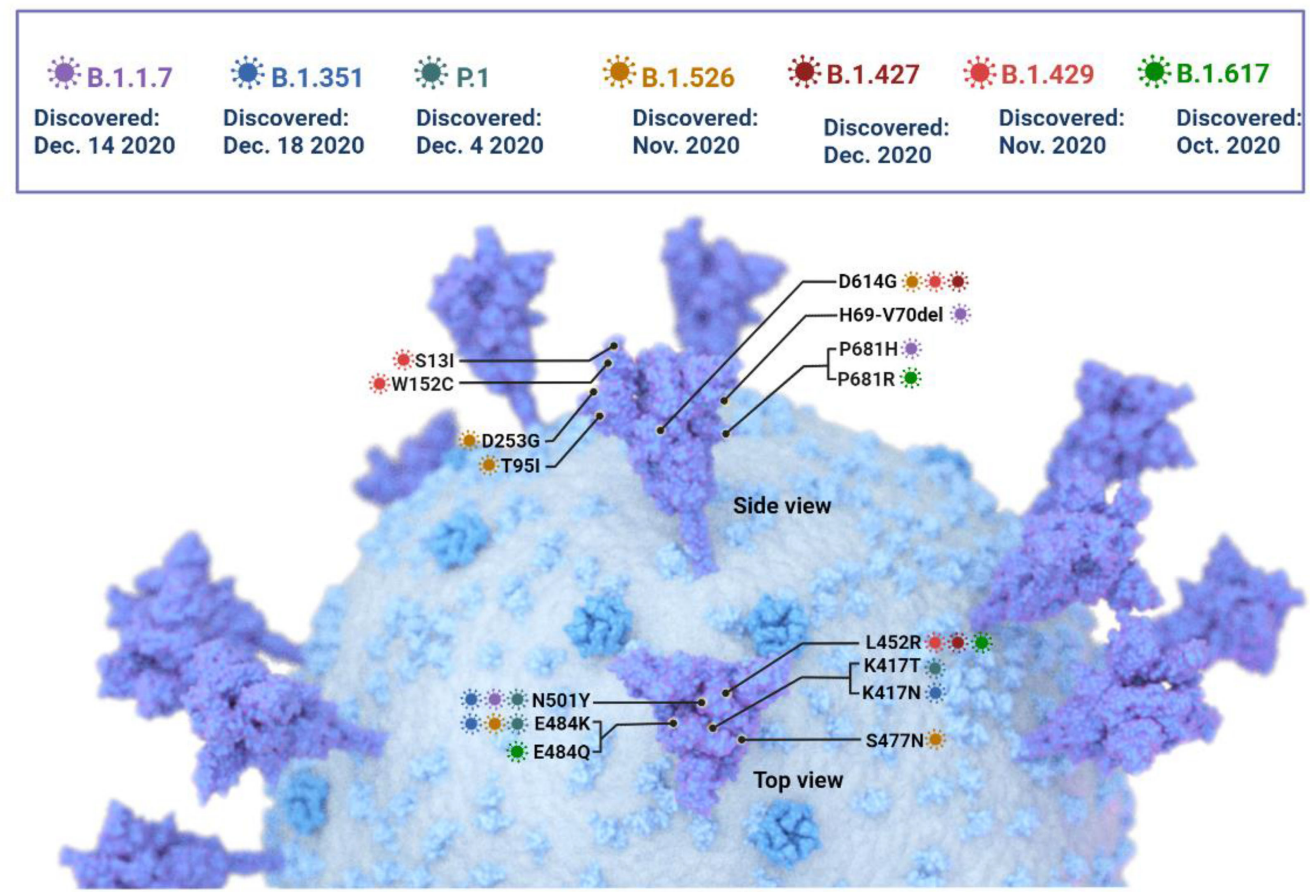
The SARS -CoV-2 Variants of concern: Key mutations in the spike protein are shown, but mutations in other areas of the genome have been identified and are currently under investigation.

SARS-CoV-2 is susceptible to genetic modification which in multiple variants has changed the potential transmission mechanism and transmission rate as shown in **Figure 3**. SARS-CoV-2 variants have been increased in rate of infection, hospitalization, and mortality (Chu et al., 2020; Tregoning et al., 2021). Multiple mutations have been observed in the spike protein and other areas of the genome as shown in **Figure 4** (Garcia-Beltran et al., 2021). The B.1.1.7 genomic variant of SARS-CoV-2 has a significant transmission advantage, the R0 (Reproduction Numbers) ranging from 0.4 (B.1.1.7) to 0.7 (non-B.1.1.7) variants. B.1.1.7 variants have been identified with a mutation in the region of the viral spike protein in the RBD (receptor-binding domain) at the global level (Wang et al., 2021; Abu-Raddad et al., 2021). Genomic sequencing of SARS-CoV-2 viral samples is essential to control the pandemic and it helps in the identification of emergent genetic variants of SARS-CoV-2 (Konings et al., 2021). The efficacy of any SARS-CoV-2 vaccine may change among the notable variants reported during this pandemic, are as shown in **Table 2**.

**Table 2:** Efficacy of SARS-CoV-2 vaccine and effectiveness on variants.

Name of Vaccine with manufacturer	Type of Vaccine	Administration Of Clinical trial	Sample size of clinical trial	Efficacy	Endpoint Analysis	Admissibility	Phase III trial with follow up	Circulating genotypes	Disease severity	References
BnT162b2 (Pfizer–BioNTech)	mRNA	2 doses (21 days apart)	43,548	95%	Positive case tested by RT–PCR and Symptomatic COVID-19 and	>16 years old	Up to 24 months after second dose (NCT04368728)	B.1.351, P.1, B.1.427/B.1.419, P.2 and B.1.526	95.3% effectivity was observed As per FDA-guideline	Lopez Bernal et al., 2021
mRnA-1273 (Moderna)	mRNA	28 days apart (2 doses)	30,420	94%	Symptomatic COVID-19	≥18 years (NCT04649151) and 6 months to 12 years (NCT04796896)	Up to 24 months after second dose (NCT04470427)	B.1.526 and B.1.427/B.1.429	More than 95% Efficacy against severe disease	Baden et al., 2021
AZD1222 (AstraZeneca University of Oxford)	Viral vector	<6 weeks apart (2 doses)	17,178	55%	Symptomatic COVID-19	≥18 years Age, ≥40 years old and not pregnant in the UK	24 months (NCT04516746)	B.1.1.7, B.1.351, P.1, B.1.427/B.1.429, P.2, B.1.526 and C.37	More than 95% efficacy against hospitalization	Voysey et al., 2021; Emary et al., 2021
		2 doses (>12 weeks		81% (Pooled efficacy 67%)	NAAT result		12 months after second dose (NCT04400838, NCT04536051 and NCT04516746)			
Ad26.CoV2-S (Johnson & Johnson)	Viral vector	1 dose	44,325	66%	Tested Positive cases	≥18 years old	25 months (NCT04505722) and 27 months (NCT04614948) after the first dose	B.1.351, P.1, B.1.427/B.1.429, P.2, B.1.526 and C.37	85.4% efficacy against severe cases	Sadoff et al., 2021
Sputnik V (Gamaley)	Viral vector	21 days apart (2 doses)	19,866	92%	Tested Positive cases	≥18 years old	6 months after the first dose (NCT04656613 and NCT04642339)	No variants have been observed in the trial region up to June 2021.	No data available up to July	Logunov et al., 2021
Covaxin (Bharat Biotech)	Viral vector	28 days apart (2 doses)	25,800	25,800 78%	Symptomatic COVID-19 and positive RT–PCR test result at least 14 days after second dose	≥18 years old (2–18 years old: study ongoing)	12 months after second dose (NCT04641481); pediatric cohort followed up for 9 months (NCT04918797)	B.1.617.2 and B.1.617.1	More than 95% efficacy against hospitalization	Yadav et al., 2021
CoronaVac (Sinovac Biotech)	Inactivated virus	14 days, (2 doses)	2,300 (Chile) 13,000 (Turkey), 12,688 (Brazil)	Various investigations; Brazil 50.7% Chile (56.5%), Turkey (91%) Indonesia, (65%), and Brazil (78%)	Tested Positive cases	≥18 years old	12 months after the first dose	P.1 and P.2	51% efficacy SARS-CoV-2, More than 95% efficacy against hospitalization infection; from 14 days after vaccination	Tanriover et al., 2021
BBIBP-CorV (Sinopharm)	Inactivated virus	2 doses (21 days apart)	45,000	78%	Occurrence of COVID-19	≥18 years old	12 months after the first dose (NCT04510207)	No variants have been identified	79% efficacy against hospitalization	Xia et al., 2021
NVX-CoV2373 (Novavax)	Protein subunit	21 days apart (2 doses)	>15,000	89%	COVID-19 positive at least 7 days after second dose	≥18 years, study ongoing, NCT04611802)	24 months after the first dose (NCT04611802)	B.1.1.7, B.1.351, B.1.427/B.1.429 and B.1.526	More than 95% efficacy against hospitalization infection	Heath et al., 2021
EpiVacCorona (VECTOR)	Protein subunit	2 doses (21–28 days apart)	3,000	Data not available July 2021	Tested Positive cases, 6 months after the first dose	≥18 years old	9 months after the first dose (NCT04780035)	No variants have been identified	No data	Doroftei et al., 2021

#### 3.2.1 D614G Variant

Spike D614G variant became prevalent at the global level within a few months after observation of original strain, D614G Variant high affinity with human ACE2 receptor in comparison to the parental strain. The D614G mutation was able to increase replication capacity and susceptibility in both human and animal models and high disease severity was observed in a patient with the D614G variant (Korber et al., 2020; Plante et al., 2021). A high level of neutralizing antibodies was analyzed in animal models with the D614G variant, and compared with the parental strain (Chen LL et al., 2021; Ozono et al., 2021). Vaccine and therapeutics agents are less effective against D614G (Korber et al., 2020). TheD614G in the Furin binding is prominent common mutations described in nearly all the new variants (Ozono et al., 2021).

#### 3.2.2 Alpha (B.1.1.7) Variant

In Dec 2020, B.1.1.7 (Alpha) of SARS-CoV-2 was reported in the United Kingdom (Oude Munnink et al., 2021). Seventeen mutations were observed in the viral genome, of which eight mutations were in the spike (S) protein such as Δ69-70 deletion, Δ144 deletion, N501Y, A570D, P681H, T716I, S982A, D1118H. Another important mutation N501Y has shown an increased severity of the infection and high binding affinity of the spike protein to ACE 2 receptors, enhancing the viral attachment and subsequent entry into host cells (Cascella et al., 2021). This variant was observed in the UK for the first time in September 2020, and in December 2020 in the USA (Galloway et al., 2021). The mortality rate was observed to be high in B.1.1.7 variant infected patients and the adjusted hazard ratio was analyzed as 1.67, 95% CI 1.34-2.09 (Cascella et al., 2021). The B.1.1.7 dominant variant SARS-CoV-2 strain is circulating in various countries globally (Port et al., 2021). BNT162b2-immunized individuals with B.1.1.7 mutations was observed a substantial reduction in neutralization titters (Emary et al., 2021; Kernéis et al., 2021). Persons immunized with Ad26.COV2-S observed a neutralization effect against the B.1.1.7 variant *in-vitro*, but was less effective than against the reference strain (Emary et al., 2021). NVX-CoV2373 SARS-CoV-2 vaccine was investigated and observed more effective (86%) against B.1.1.7 variant in comparison to the original strain (96%). The phase III clinical trial was evaluated with 15,000 participants (18 and 84 years of age) in the UK (Galloway et al., 2021; Shinde et al., 2021). The efficacy of AZD1222 was observed 70% in patients with B.1.1.7. In the case of non-B.1.1.7 Lineages, 77% efficacy was observed (Lopez Bernal et al., 2021). Various amino acid modifications have been observed in the spike protein of B.1.1.7. including P681H, N501Y, 69/70, E484K, and ORF8 mutations (Collier et al., 2021; Kernéis et al., 2021; Seghatchian, 2021). Another mutation F888L in the spike protein was also identified in the Nigerian variant along with the E484K (Port et al., 2021; Ashoor et al., 2021). This mutation has been reported to alter the biological efficiency of SARS-CoV-2 by performing the hydrolysis by TMPRSS2 and augmenting viral invasion (Ashoor et al., 2021; Liu C et al., 2021). It is also probable that this major variation supports the viruses’ misleading of the immune response of the host (Kernéis et al., 2021; Liu J et al., 2021; Quinonez et al., 2021). All the above investigations were carried out with their limitations in respect to methodology, sample size, and immune response (Kernéis et al., 2021).

#### 3.2.3 Lineage B.1.351 (Beta)

The B.1.351 variants were reported in South Africa in December 2020 (Shinde et al., 2021). B.1.351 variants carry E484K mutations and cause more severe symptoms than the other variants (Hoffmann et al., 2021). Emergent mutant variants from the UK and South Africa are more infectious, but effective against developed vaccines (Voysey et al., 2021). The 501.V2 variants was the first time observed in South Africa, it carries K417N and E484K mutations. The 501.V2 are more transmissible and cause severe in comparison to the parental strain (Wang et al., 2021). E484K mutation also plays an important role in immune mechanism, host receptor affinity, and infectivity (Shastri et al., 2021). Initial findings have indicated that the Oxford–AstraZeneca vaccine has shown considerable reduction in effectiveness against these variants and was reviewed by the WHO (Lopez Bernal et al., 2021). Novavax can protect up to a moderate level, while the Pfizer–BioNTech and Johnson & Johnson vaccines also have reduced the efficacy against the β-lineage, although for the other vaccines the data is not yet available (Heath et al., 2021; Sadoff et al., 2021; Lopez Bernal et al., 2021). It has also been identified that the most extensively distributed vaccines may have reduced antibody neutralization against Beta variants of SARS-CoV-2, which is relevant to how vaccines can stop the disease by reducing asymptomatic infection (Grint et al., 2021). Sera from patients immunized with the Moderna and Pfizer-BioNTech vaccines had less activity against Beta (B.1.351) (Bates et al., 2021). On 1 April 2021, an investigation on a Pfizer/BioNTech South African vaccination analysis showed that the vaccine has been 100% effective against Beta variants (Seppälä et al., 2021). In January 2021 Ad26.COV2. S Vaccine developed by Johnson & Johnson has been tested in South Africa and reported 72% efficiency against SARS-CoV-2 infection (mild to severe) in the US and 57% in South Africa (Shinde et al., 2021; Voysey et al., 2021).

#### 3.2.4 Cluster 5 Variant

The Cluster 5 Variant may decrease the strength of immune defenses, after a decrease in the viral neutralization sensitivity which is obtained by vaccination and the normal procedure of infection (Becker et al., 2021). In Denmark, ΔFVI-spike was categorized as Cluster 5 strains transmitted from mink worsened the situation and may further aggravate it. As of November 2020, active mink-mediated corona cases are confirmed (Lassaunière et al., 2021). As per WHO data, the cluster 5 variant resisted diminishing sensitivity to countering antibodies. Viral expansion in mink lakes leads to a recurring risk of human infection from mink, and the adaptability of this variant in mink is a huge health concern in the future (Hoffmann et al., 2021).

#### 3.2.5 Lineage B.1.258Δ

This variant was identified in the Czech Republic and Slovakia in late 2020, within the clade B.1.258 (Brejová et al., 2021). It has been observed to escape the immune response and increase the severity of infection. This variant has been analyzed with an N439K mutation in the terminal regions of the spike glycoprotein while showing similar deletions 69-70 at the receptor-binding domain (RBD) (Gómez et al., 2021). H69/V70 deletions mutations have been modified. The antigenic peptides in the amino-terminal region are changed, subsequent in confrontation to neutralization by improving sera and vaccination (Jeong et al., 2021).

#### 3.2.6 P.1 or 20 J/501Y.V3 Variants

A new variant has been observed in Lineage P.1 with 11 mutations in the spike protein. Since December 2020, approximately 42% of SARS-CoV-2 positive tested samples were analyzed with P.1 lineage infection (Hoffmann et al., 2021). These mutations are closely linked with antibody-mediated immune evasion high infection rate (+161%), the mortality rate was also observed to be high, up to 50%, making it 2.2 times more transmissible than the baseline virus (Sarkar et al., 2021). P.1 and P.1-like clades are more infective in younger people. P.1 or 20 J/501Y.V3 were classified as gamma mutations (K417T, E484K, and N501Y) in the RBD domain (Hirotsu and Omata, 2021). P.1 or 20 J/501Y.V3 variants with E484K substitution were reported in Brazil (Hirotsu and Omata, 2021). In November 2020 and January 2021, it has been observed that the Gamma variant is 1.4-2.2 times more infectious than baseline (Hitchings et al., 2021). People who have been completely vaccinated with Pfizer or Moderna have shown significant neutralization against Gamma variants (Planas et al., 2021). The data from various clinical trials carried out by the WHO, CoronaVac, and BBIBP-CorV shows effectiveness against Gamma variants (Barros-Martins et al., 2021). They also found that Oxford–AstraZeneca, and CoronaVac had preserved antibody neutralization against Gamma linage, and Pfizer–BioNTech and Moderna had minimal to moderate reduction in neutralization, with no information for other vaccines so far (Barros-Martins et al., 2021).

#### 3.2.7 Lineage B.1.617 and B.1.617.2

Three major substitution mutations were observed in Lineage B.1.617 and B.1.617.2, namely P681R, L452R, and E484Q (Liu J et al., 2021). Two substitution mutations were observed in the RBD domain, and one nearby the furin binding the site, which increases the mode of transmission (+64%), hospitalization (+85), and mortality, with natural immunity also affected. The chances of reinfection were decreased, but the efficacy of the vaccine was also compromised in Lineage B.1.617 and B.1.617.2 (Lopez Bernal et al., 2021). Various investigations were examined by the WHO and found that vaccines from Oxford–AstraZeneca, and Pfizer–BioNTech, are likely to sustain efficacy/effectiveness against delta’s variants. (Liu J et al., 2021). Researchers from the field also investigated and found that the vaccines produced by Oxford–AstraZeneca have shown a reduced neutralization effect against the Delta virus (Pascarella et al., 2021). Spike protein mutations D111D, E484Q, G142D, and P681R are found in the delta variants of 15 mutations, which may escape antibody neutralization. Initial observations have shown that Emergent variants reduce the efficacy of the mRNA-based vaccine (Cherian et al., 2021). Researchers from the field also explored whether the E484K variant may compromise the efficiency of the current vaccine. More clinical investigation is required to finally reach a conclusion for therapeutic strategies against new variants.

#### 3.2.8 Other Variants

Lineage B.1.168 was observed in West Bengal, India with two amino acid deletions, Tyr145 and His146, and E484K and D618G mutations (Cherian et al., 2021). These substitutions can escape convalescent plasma and multiple monoclonal antibodies (Bates et al., 2021). Other 1.5.9 variants are also reported in different countries (Singh et al., 2021). The B.1.429 or Epsilon lineage was reported in 50% of samples in Los Angeles, which exhibits different mutations in ORF1ab and spike protein (Shen et al., 2021). The variants B.1.429 and B.1.427 were classified as VOCs by the CDC (Shen et al., 2021). These variants with D1183Y and I4205V mutations in the ORF1ab and S13I, W152C, and L452R mutations in the spike protein are also known as CAL.20C, 20C/S:452R, CA VUI, or 21C. CAL.20C variants were identified in November 2020 in California, this variant was classified Epsilon (McCallum et al., 2021). The prevalence of the variant in sequenced samples from Northern California increased from 3% to 25% between November and December 2020 in total samples tested in California (Zhang et al., 2021). In January 2021, the prevalence of the variant 20G was observed in the United States. This variant was also seen in some SARS-CoV-2 cases in Europe, Asia, and Australia (McCallum et al., 2021). The frequency of this variant was decreased in February 2021, until April 2021 by when this Epsilon variant had disappeared from southern California and comprised just 3.2 percent of cases were observed in the United States, while 2/3 cases were observed from Alpha variants (Zhang et al., 2021). Theta variants (P.3.) were reported on February 18, 2021, with two mutations E484K and N501Y, by the Central Visayas Department of the health service of the Philippines (Oude Munnink et al., 2021). Theta variants (theta) were also identified in Japan, the United Kingdom, and Malaysia in July 2021. Theta variants disappeared by July 2021. Another, the R.1 variant was reported by Japan on the RBD of the spike protein with E484K mutation, and another W152L mutation was observed in the N-terminal Domain. These mutations also play an important role in immune evasions, and are reported by various countries at the global level (Chen RE et al., 2021). The Pfizer-BioNTech vaccine was shown to be 94% effective in preventing R.1 infected hospitalization and mortality. As Alpha and then Delta increase in Japan, R.1 illustrations are becoming increasingly rare (Kernéis et al., 2021). Linage B.1.620 was analyzed in Lithuania in March 2021, also known as the Lithuanian strain, it was found in Central Africa, North America, France, and Belgium. In an analysis of the original variant, this lineage revealed 23 substitutions, most of which are discrete mutations (Silva et al., 2021; Korber et al., 2020). Lineage B.1.618 was discovered for the first time in October of 2020, this variant contained E484K mutation with many other variations and showed substantial development in West Bengal, India, in April 2021 (Biswas et al., 2021). On 23 April 2021, The PANGOLIN analysis identified 135 sequences in India (Singh et al., 2021). Sixteen cases in the United Kingdom were identified under Lineage B.1.1.318 as a VUI (VUI-21FEB-04) (Seghatchian, 2021).

## 4 Conclusion

Researchers from the field are focused on the eradication of SARS-CoV-2 infection from serious health risks to humans. In this regard various clinical approaches and scientific methods are exponentially used against SARS-CoV-2 from virtual drug screening to the molecular mechanism, and from vaccine designing to SARS-CoV-2 platforms development, computational approaches are of great interest. They have enhanced the understanding of genomics designs, proteomics, structures determination, mutation solidity, function connection, and tracing. There are now enough investigations of the altering antigenicity of the SARS-CoV-2 spike protein and of the amino acid variations that can change antibody neutralization. Spike amino acid substitutions and deletions influence neutralizing antibodies efficacy in the global virus population. However, our knowledge about SARS-CoV-2 is very limited. No effective treatment option and anti- SARS-CoV-2 approaches are have reached their final design. Further clinical investigation is required to prevent infection and control the pandemic situation at the global level.

## Author Contributions

D.D.S. and D K Y. conceived and designed the project, D.D.S., A.P. and D K Y. collected data from the literature. D.D.S. analyzed the data and wrote the manuscript. All authors have read and approved the final version of the manuscript. Figure was created using BioRender (https://biorender.com/ accessed on 16th July 2021).

## Funding

D.K.Y. and A.P. are thankful to the Basic Science Research Program of the National Research Foundation of Korea (NRF), funded by the Ministry of Education, Science, and Technology, who supported this study (No. 2017R1C1B2003380 and NRF2019R1G1A1003693).

## Conflict of Interest

The authors declare that the research was conducted in the absence of any commercial or financial relationships that could be construed as a potential conflict of interest.

## Publisher’s Note

All claims expressed in this article are solely those of the authors and do not necessarily represent those of their affiliated organizations, or those of the publisher, the editors and the reviewers. Any product that may be evaluated in this article, or claim that may be made by its manufacturer, is not guaranteed or endorsed by the publisher.
