# Corrigendum: SARS-CoV-2: Emergence of New Variants and Effectiveness of Vaccines

**DOI:** 10.3389/fcimb.2022.885482

**Published:** 2022-06-01

**Authors:** Desh Deepak Singh, Amna Parveen, Dharmendra Kumar Yadav

**Affiliations:** ^1^ Amity Institute of Biotechnology, Amity University Rajasthan, Jaipur, India; ^2^ Gachon Institute of Pharmaceutical Science and Department of Pharmacy, College of Pharmacy, Gachon University, Incheon, South Korea

**Keywords:** SARS-CoV-2, variant, vaccine, neutralization, infectivity

Due to some error in the published paper, the author order needs to be corrected.

The correct sequence of authors is: Desh Deepak Singh, Amna Parveen and Dharmendra Kumar Yadav. 

The authors apologize for this error and state that this does not change the scientific conclusions of the article in any way. The original article has been updated.

## Publisher’s Note

All claims expressed in this article are solely those of the authors and do not necessarily represent those of their affiliated organizations, or those of the publisher, the editors and the reviewers. Any product that may be evaluated in this article, or claim that may be made by its manufacturer, is not guaranteed or endorsed by the publisher.

